# Metabolic Reprogramming of Colorectal Cancer Cells and the Microenvironment: Implication for Therapy

**DOI:** 10.3390/ijms22126262

**Published:** 2021-06-10

**Authors:** Miljana Nenkov, Yunxia Ma, Nikolaus Gaßler, Yuan Chen

**Affiliations:** Section Pathology of the Institute of Forensic Medicine, University Hospital Jena, Friedrich Schiller University Jena, Am Klinikum 1, 07747 Jena, Germany; miljana.nenkov@med.uni-jena.de (M.N.); Yunxia.ma@med.uni-jena.de (Y.M.); Nikolaus.gassler@med.uni-jena.de (N.G.)

**Keywords:** metabolism, colorectal cancer, the tumor microenvironment, intestinal microbiota, CRC therapy

## Abstract

Colorectal carcinoma (CRC) is one of the most frequently diagnosed carcinomas and one of the leading causes of cancer-related death worldwide. Metabolic reprogramming, a hallmark of cancer, is closely related to the initiation and progression of carcinomas, including CRC. Accumulating evidence shows that activation of oncogenic pathways and loss of tumor suppressor genes regulate the metabolic reprogramming that is mainly involved in glycolysis, glutaminolysis, one-carbon metabolism and lipid metabolism. The abnormal metabolic program provides tumor cells with abundant energy, nutrients and redox requirements to support their malignant growth and metastasis, which is accompanied by impaired metabolic flexibility in the tumor microenvironment (TME) and dysbiosis of the gut microbiota. The metabolic crosstalk between the tumor cells, the components of the TME and the intestinal microbiota further facilitates CRC cell proliferation, invasion and metastasis and leads to therapy resistance. Hence, to target the dysregulated tumor metabolism, the TME and the gut microbiota, novel preventive and therapeutic applications are required. In this review, the dysregulation of metabolic programs, molecular pathways, the TME and the intestinal microbiota in CRC is addressed. Possible therapeutic strategies, including metabolic inhibition and immune therapy in CRC, as well as modulation of the aberrant intestinal microbiota, are discussed.

## 1. Introduction

Colorectal carcinoma (CRC) is the third most common cause of cancer death in both males and females in the United States, and globally, it ranks as the world’s fourth most deadly cancer, with almost 900,000 deaths annually [1,2]. Although progress in the battle against CRC has been made over the past few decades due to more widely applied screening tests, including stool tests, colonoscopy and blood-based biomarker tests, as well as development of personalized targeted therapy and immunotherapy, the mortality and morbidity of CRC are still increasing. According to the SEER database (https://seer.cancer.gov/statfacts/html/colorect.html, accessed on 9 June 2021), the 5-year survival rate for CRC patients with all stages included is 64.4% in the United States [3]. An alarming tendency of increasing CRC incidence in younger patients (less than 50 years old) has been observed, particularly for those between the ages of 40 and 49 years [4]. The reasons for early-onset of CRC may be associated with several factors, including smoking, increased dietary fat uptake and obesity in younger adults [5].

Approximately only 5% of CRC cases are hereditary, closely related to Lynch syndrome (hereditary nonpolyposis colorectal cancer), while the majority of CRC cases are sporadic and develop slowly [6]. Colorectal tumorigenesis is believed to be a multiple-step process, which has been considered an adenoma–carcinoma sequence and may take several years to accomplish [7]. Colorectal tumorigenesis goes through a series of genetic and epigenetic alterations, including somatic mutations, chromosomal instability, microsatellite instability and DNA methylation, as well as histone acetylation, leading to the activation of oncogenes and the inactivation of tumor suppressor genes [8,9]. Mutation of the tumor suppressor gene APC is detectable in 70% of benign colorectal adenomas, resulting in the inactivation of APC in the majority of colorectal adenomas. Followed by activation of oncogene KRAS due to mutation and the inactivation of the tumor suppressor gene p53, PTEN and SMAD4, most of the colorectal adenomas ultimately develop to CRC [8]. Recently, it has been suggested that CRC may be classified into four different consensus molecular subtypes (CMS) based on gene expression data [10,11]. The distinct characteristics of the four CMS reflect the heterogeneity of CRC as well as its unique clonal, stromal, immune and metabolic associations [12]. 

Metabolic reprogramming contributes to tumor progression and metastasis and is considered an important hallmark of cancer. In order to meet their high demand for energy and nutrients, cancer cells usually rewire metabolism to support their rapid cell division. In different types of cancers including CRC, dysregulated metabolic pathways have been frequently observed, resulting in abnormal glycolysis, glutaminolysis and lipid synthesis [13]. While driver mutations in oncogenes and tumor suppressor genes govern metabolic programs, they also influence gene expression and epigenetic regulation, and reshape the tumor microenvironment (TME) and the gut microbiota [14]. The crosstalk between tumor metabolites and TME components as well as the intestinal microbiota is tightly related to therapy effectiveness in cancer [15]. It is also worth to mention that the metabolic reprogramming takes place even at the colon adenoma stage [16].

In this review, we depict the abnormal metabolic pathways and their impact on the development and progression of CRC. Additionally, the metabolic crosstalk between cancer cells, the TME and the gut microbiota in the progression of CRC is addressed. A better understanding of the metabolic network in CRC will lead to the development of a novel therapeutic strategy in the management of this fatal disease.

## 2. Metabolism

Nutrients including carbohydrates, fatty acids and amino acids, essential for cell homeostasis and macromolecular synthesis in humans, are processed through anabolic or catabolic pathways [17]. Catabolic pathways (catabolism) produce energy by breaking down the nutrients, while anabolic (anabolism) are synthesis reactions of complex macromolecules from simple ones [17]. Metabolism, including both catabolism and anabolism, is essential for nutrient utilization and energy production in living organisms [17]. Abnormalities in cellular metabolism have been reported in many types of cancers.

### 2.1. Warburg Effect

The first evidence of the metabolic distinction between normal and cancer cells was proposed in 1924 by Otto Heinrich Warburg [18]. The introduced term “the Warburg effect” refers to the preference of cancer cells to use glycolysis to produce energy in the form of ATPs (adenosine triphosphate) over oxidative phosphorylation (OXPHO), even in the case of sufficient oxygen supply [18]. The observation of “the Warburg effect”, also called aerobic glycolysis, led to the introduction of a new hallmark of cancer, the reprogramming of the cell metabolism [19]. This metabolic reprogramming, aerobic glycolysis, provides highly proliferative cancer cells with glycolytic intermediates used for biosynthesis of biomolecules, such as lipids, nucleotides and amino acids, which are essential for supporting cancer cell rapid growth, division and survival [20,21]. 

The Warburg effect was reported in many different cancers, including colorectal cancer. Interestingly, studies showed an upregulation of HIF-1α (hypoxia-inducible factor 1 α), GLUT1 (glucose transporter 1), PKM2 (pyruvate kinase M2) and LDHA (lactate dehydrogenase A 1) involved in glycolysis in precancerous colorectal lesions compared to normal control, indicating that the Warburg effect occurs even in the premalignant tissues [22].

### 2.2. Glucose Metabolism

**Figure 1:**
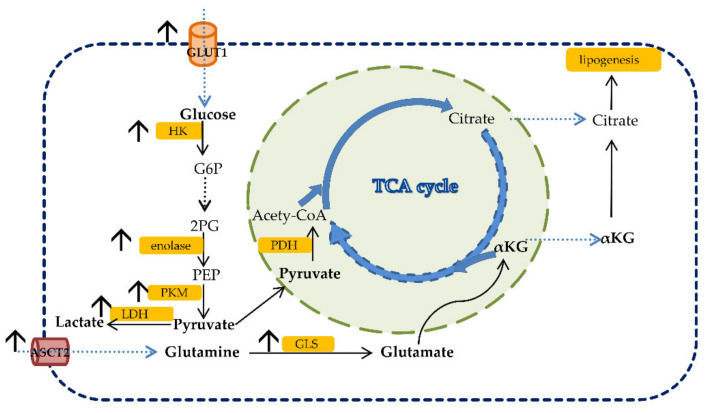
Altered glucose and glutamine metabolism in CRC. Dysregulated enzymes are represented with ↑ indicating upregulation. Glucose is uptaken by the cell through GLUT1 transporter and is further metabolized by different enzymatic reactions. Cancer cells are favoring lactate accumulation through upregulating enzymes such as LDH. Detailed information is provided in the text.2PG: 2-phosphoglycerate; PEP: phosphoenolpyruvate; PDH: pyruvate dehydrogenase.

Glucose is initially uptaken by cells via glucose transporters (GLUTs). GLUT1 is increased in most cancers, and it is associated with a worse prognosis [21]. Glucose is phosphorylated to glucose-6-phosphate (G6P) by hexokinase (HK). HK2, located on the outer membrane of mitochondria, is increased in cancers. Phosphofructokinase (PFK) catalyzes the phosphorylation of F6P to fructose-1,6-biphosphate (F1,6BP), and it is positively regulated by PI3K via the induction of glycolytic flux. Pyruvate kinase (PK) regulates the reaction and the final step of glycolysis [21]. Abnormal expression of PKM2 (PK muscle isozyme M2) was observed in many cancers including CRC [23,24,25]. In the case of oxygen absence, the production of pyruvate in glycolysis is converted to lactate in the fermentation reaction by lactate dehydrogenase (LDH). Increased lactate levels and overexpression of GLUT1, pyruvate kinase M2, glyceraldehyde-3-phosphate dehydrogenase, enolase-1α, lactate dehydrogenase 5 and hexokinase 2 have been found in CRC [26,27,28,29,30,31,32]. Some of the tricarboxylic cycle (TCA) enzymes were found to be mutated in cancers associated with poor prognosis, including CRC. For example, succinate dehydrogenase (SDH) is an enzyme responsible for converting succinate into fumarate. Mutations of SDH lead to the stabilization of HIF-1α and, consequently, hypermethylation of DNA and histones, related to tumor progression [33]. The alteration in glucose metabolism in CRC is shown in Figure 1.

### 2.3. Glutamine, Serine and Tryptophan Metabolism

Glutamine belongs to the group of non-essential amino acids, and it is the most prominent amino acid in human plasma [21]. The non-essential term for glutamine may switch to conditionally essential, depending on the demand of the cells that need glutamine in the case of extremely rapid cell growth. Glutamine is transported into the cell cytosol via the Na+ dependent antiporter of neutral amino acids, and it is the preferred substrate of SLC1A5, also called ASCT2 (alanine, serine, cysteine transporter 2) [34,35]. SLC1A5 is upregulated in many cancers, including CRC [36,37,38]. In the study by Huang et al., overexpression of SLC1A5 was found to stimulate CRC cell growth and survival [39]. Glutamine is metabolized through a process called glutaminolysis. Glutamine is firstly deaminated into glutamate using glutaminase (GLS), which is further converted into α-ketoglutarate (α-KG) and used for the synthesis of macromolecules. Rapidly proliferating cancer cells use high amounts of glutamine to facilitate their survival [40]. The α-ketoglutarate (α-KG) generated during glutaminolysis can be metabolized into citrate during reductive phosphorylation. Citrate is then converted into acetyl-CoA, which is used for lipid synthesis (Figure 1). Serine, also a non-essential amino acid, contributes to the one-carbon metabolism. CRC cells have been found to rely on serine for their proliferation [41,42,43,44]. Tryptophan, an essential amino acid, is processed through two pathways generating either NAD+ or serotonin [45,46]. The enzyme responsible for the conversion of tryptophan to kynurenine, named indole-amine (2,3)-dioxygenase (IDO), is overexpressed in cancers including CRC and is associated with tumor progression and the overall survival of patients [47].

### 2.4. One-Carbon Metabolism

**Figure 2:**
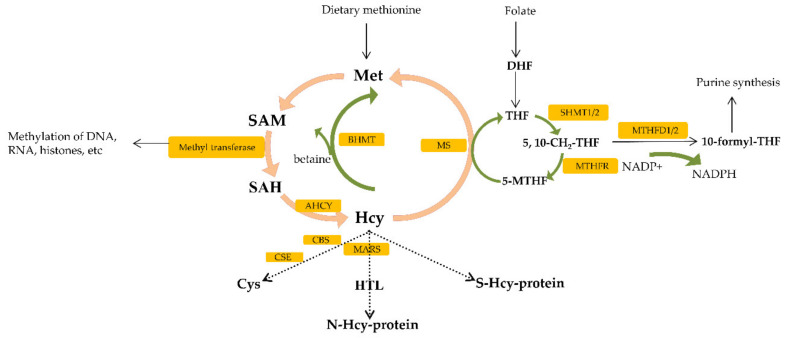
The folate and methionine cycle of one-carbon metabolism, as described in the text in detail.

One-carbon metabolism (1C), by coupling both the folate and methionine cycle, generates 1C groups that are further used for the biosynthesis of essential precursors such as purines, pyrimidines and methylation reactions [48]. Folate, an essential vitamin belonging to the group of vitamin B9, needs to be taken from the diet and acts as a carrier of 1C-group, shuttling this group from the input intermediates to the output [41]. Dietary folate can be converted through the cycle into several metabolites, each of which has a distinct role in metabolism [49]. Three most important metabolites used for different biosynthesis reactions are as follows: 5-methyl tetrahydrofolate (THF), 10-formyl THF and 5,10-methylene THF [49]. The central step in the folate cycle is conversion of serine to glycine, catalyzed by the serine hydroximethyl transferase1 (SHMT1) (cytosol) and serine hydroximethyl transferase 2 (SHMT2) (mitochondria) [49] (Figure 2). Serine serves as carbon donor to initiate the folate cycle, converting THF to 5,10-methylene THF [14]. Then, 5,10-methylene THF can be reduced to 5-methyl-THF by methylenetetrahydrofolate reductase (MTHFR). 5-methyl-THF donors 1C-atom to homocysteine in methionine cycle reaction [14]. 5,10-methylene THF can be also converted into 10-formyl THF via methylenetetrahydrofolate dehydrogenase 1 (MTHFD1) (cytosolic) or via mitochondrial coupled enzymes methylenetetrahydrofolate dehydrogenase (MTHFD2L/MTHFD2) and used for purine synthesis reaction [49] (Figure 2).

Increased expression of folate-dependent one-carbon metabolic enzymes, including, folate receptor-1 (FOLR1), dihydrofolate reductase (DHFR), serine hydroxymethyltransferase 1 (SHMT1), serine hydroxymethyltransferase 2 (SHMT2) and methylenetetrahydrofolate reductase (MTHFR) was observed in CRC cells compared to untransformed cells [50]. SHMT2 was found to be significantly increased in a variety of cancers, including colorectal cancer [51]. In the study by Wei et al., SHMT2 overexpression was reported, and it was associated with poor survival among CRC patients [52]. Posttranslational modification of SHMT2 by lysine acetylation in the case of high glucose presence is associated with SHMT2 destabilization and, furthermore, TRIM21-mediated lysosomal degradation [52]. In CRC cells, SHMT2 is stabilized through its SIRT3-mediated deacetylation [52]. A high expression level of MTHFD1L is also reported in patients with primary CRC, and downregulation of MTHFD1L suppressed the colon cancer cell proliferation and invasion rate [53]. Expression of the one-carbon metabolism enzymes SHMT2, MTHFD2 and mitochondrial 10-formyltetrahydrofolate dehydrogenase (ALDH1L2) was found to be highly upregulated in CRC tissues when compared to the normal control [54].

Dietary methionine is processed through multiple reactions called the methionine cycle [55]. Firstly, methionine (Met) is converted to S-adenosylmethionine (SAM) which is known as a methyl donor for DNA, histone and non-histone proteins [50] (Figure 2). Upon SAM-mediated transmethylation reaction, S-adenosylhomocysteine (SAH) is generated [55,56]. SAH is hydrolyzed to homocysteine (Hcy) by SAH hydrolase (AHCY); this reaction is the main and only source of Hcy in human body [55,57]. Hcy is further metabolized in cell via the following pathways such as remethylation to methionine by Met synthase (MS) or betaine: Hcy methyltransferase (BHMT), transsulfuration to cysteine (Cys) by cystathionine β-synthase (CBS) and cystathionine γ-lyase (CSE) and conversion to Hcy-thiolactone (HTL) by Met-tRNA synthetase (MARS) [58]. Additionally, Hcy that is transported from cell reacts in plasma, with proteins-thiol groups forming such as S-Hcy-protein, homocystine (Hcy-S-S-Hcy) and a Cys-S-S-Hcy disulfide [55,58]. Since Hcy is generated as a byproduct of cellular methylation reactions, it represents a sensitive marker of one-carbon metabolism as well as epigenetic processes [55,59,60]. Epigenetic mechanism is important for regulating gene expression through DNA methylation, histone modification and noncoding RNA activity [55].

Dysregulation in homocysteine metabolism, precisely hyperhomocysteinemia (HHcy), has been associated with a variety of diseases including cancer [61] Hcy and its metabolites including Hcy-thiolactone and *N*-Hcy-protein have been related to cancer in previous studies [62]. Changes in gene expression induced by Hcy, Hcy-thiolactone and *N*-Hcy-protein have been found to contribute to the dysregulated one-carbon metabolism in cancer [62]. HHcy results from accumulated levels of Hcy and its metabolites, including, HTL, *N*-Hcy-proteins, S-Hcy-proteins, Hcy disulfides and SAH [55,63]. SAH, generated after SAM-mediated donor methyl reaction, acts as an inhibitor of methylation. HHcy through elevation of SAH and reduction in SAM/SAH ratio may result in global hypomethylation; however, experimental studies showed that this is not always the case [64,65]. 

Epigenetic dysregulation caused by Hcy metabolite such as HTL has been reported in research studies [66]. HTL is produced from Hcy by methionyl-tRNA synthetase (MARS) during protein biosynthesis [57]. HTL reacts with ε-amino group of a protein lysine residue forming *N*-Hcy-linked protein [57]. This post-translational modification (*N*-homocysteinylation) significantly influences protein structure and function, thus causing severe diseases [57]. Protein *N*-homocysteinylation has been reportedly involved in colorectal cancer (CRC) [55]. High fat diet has been showed to increase Hcy level in both human and animal studies related to high risk of CRC [67,68,69]. Significantly increased plasma level of *N*-Hcy has been found in CRC patients compared to healthy controls, associated with CRC progression [69]. The plasma Hcy level has not been correlated with CRC progression, indicating that *N*-Hcy-protein might be responsible for promoting CRC [69]. In addition, higher expression of *N*-Hcy-protein and MARS has been detected in CRC tissues compared to normal [69]. Several proteins involved in DNA damage repair have been modified by *N*-homocysteinylation and their function was impaired due to abnormal *N*-homocysteinylation in CRC. Particularly, ataxia telangiectasia and Rad3-related protein (ATR) was modified and consequently inactivated in CRC cell (HCT116 [69]. In addition to promoting migratory activity in cancer cells, increased K-Hcy modification elevated the microsatellite instability in cancer cells [69]. ATR K-Hcy increased DNA damage and promoted CRC cell survival and proliferation in presence of DNA damage [69]. Inhibiting the K-Hcy modification by preventing HTL synthesis catalyzed by MARS resulted in decreased DNA damage and cell proliferation, suggesting the potential of MARS inhibitors (*N*-acetyl cysteine (NAC) and acetyl-homocysteine thioether (AHT)) in CRC treatment [69]. Another pharmacological possibility is to accelerate HTL hydrolysis by enzymes such as paraoxonase 1 (PON1), bleomycin hydrolase (BLMH) or bisphenol hydrolase-like (BHPL) which will result in HTL reduction and consequently *N*-Hcy-protein attenuation [70,71].

Dietary nutrients such as methionine depleted/rich diet could affect epigenetic regulation such as histone methylation [72]. Under methionine-restricted conditions in HCT116, loss of H3K4me3 (histone methylation marks involving trimethylation at lysines 4) resulted in decreased expression of colorectal cancer (CRC)-associated genes such as AKT1, MYC and MAPK [72].

Tumors with different origins are usually associated with global hypomethylation patterns and some specific gene hypermethylation [73]. The significant contribution of serine in SAM production was reported in CRC cells with the deprivation of serine in cancer cells, leading to lower methylation of DNA and others [42]. 

### 2.5. Lipid Metabolism

Lipids are essential nutrients for cells, acting as the structural components of cell membranes, signaling molecules and energy suppliers. Lipids can be classified into several groups, but the most abundant lipids are fatty acids, triglycerides, sphingolipids, phospholipids and cholesterol [74]. Abundant lipids were detected in aggressive CRC, in line with the fact that CRC was accompanied by upregulated lipogenesis [75,76,77,78,79,80]. Long et al. identified the difference in lipid contents between adenocarcinoma and non-adenocarcinoma, with 24 lipids out of 36 differentially expressed metabolites [79]. Abnormal fatty acid metabolic pathways were reported to drive tumor development and progression, correlating with a poor prognosis in CRC patients [81,82,83,84]. Complex lipids are composed of many different molecules including fatty acids (FAs), glycolipid, glycerophospholipids, sphingolipid, etc. [82]. The most abundant dietary fatty acids are long-chain fatty acids, and they can be transported into cells via the following proteins: cluster of differentiation 36 (CD36), also known as FA translocase (FAT); plasma membrane fatty acid-binding protein (FABPpm); fatty acid transport proteins 1–6 (FATP1–6); and caveolin-1 [82,85,86]. The increased fatty acid uptake mediated by CD36 could be associated with tumor metastasis and progression in various cancers including hepatocellular, gastric, ovarian and cervical cancers [87,88,89,90]. In a colorectal cancer study, it was reported that the reduction in stroma CD36 led to upregulated vascularization [91]. Furthermore, activation of CD36 inhibited CRC cell proliferation and induced apoptosis [92]. As opposed to the primary CRC, CD36 was found to be upregulated in the metastatic lesions of CRC, implying the higher dependency of metastatic tumors on FA uptake as compared with primary CRC [93,94,95]. Fatty acid transport proteins (FATPs), the solute carrier 27 (Slc27) family of proteins, play a role in exogenous fatty acid transport, in addition to very long-chain acyl-CoA synthetase [96,97]. FATPs transport LCFA to cells and activate LCFAs through ACSVL carrier acylation. The expression of FATP2, one family member of FATPs, was associated with increased tumor growth and therapy resistance [97]. 

Fatty acid binding proteins (FABPs) are involved in fatty acid uptake and transport [98,99,100]. In addition, their roles in the regulation of gene expression, cell differentiation and growth have also been observed [101,102]. Several FABPs have been found to be highly expressed in cancer cells [98]. Upregulation of FABP5-induced CRC cell proliferation and invasion via the PPAR β/δ-independent signaling pathway [98]. FABP6 participates in bile acid transport in the ileum, and it is involved in tumorigenesis [103]. Ohmachi et al. found higher expression of FABP6 in primary CRC compared with normal control; however, FABP6 expression was significantly reduced in metastatic lymph nodes, suggesting the role of FABP6 in early colorectal carcinogenesis [103,104,105,106,107]. Unlike normal cells, cancer cells are characterized by high fatty acid de novo synthesis even when sufficient exogenous fatty acids are available [84,108,109]. Once FAs are transported into the cells via the previously mentioned mechanisms, they are activated by coupling with CoA, by the group of enzymes, long chain acyl CoA synthetases (ACSLs) and very long chain acetyl CoA synthetases (ACSVL). Concomitant overexpression of ACSL1, ACSL4 and SCD1 was found to be able to induce epithelial to mesenchymal transition (EMT) in CRC [110].

When the fatty acids are partitioned into the FAO (fatty acid oxidation pathway), they must translocate across the mitochondrial membrane [74]. Carnitine palmitoyltransferase 1 (CPT1) is responsible for the conversion of acyl-CoA into acyl carnitine by coupling to carnitine. The translocation of acyl carnitine is mediated by carnitine acyl carnitine translocase (CACT). Once delivered into mitochondria, acyl carnitine is returned to the acyl-CoA by carnitine palmitoyltransferase 2 (CPT2) [74]. In FAO, acyl-CoA is processed further, and the end product, acetyl-CoA, is then involved in the TCA cycle [74]. CPT1A showed a higher expression in metastatic than in primary CRC [94].

Citrate produced from the TCA cycle is partitioned into acetyl-CoA and oxaloacetate in the cytoplasm via ATP-citrate lyase (ACLY). Acetyl-CoA is further converted into malonyl-CoA by the acetyl-CoA carboxylase (ACCs). Then, the condensation of malonyl-CoA and acetyl-CoA is conducted by fatty acid synthase (FASN). Palmitic (C16) acid, generated by FASN, can be further elongated and desaturated by SCD (stearoyl-CoA desaturase). These key de novo fatty acid synthesis enzymes, such as ACLY, ACC, FASN and SCD, have been found upregulated in different cancers [74,111,112,113,114,115,116]. A study by Wen et al. revealed that ACLY mediates the CRC growth inhibitory effects by interacting with β-catenin, leading to the stabilization and translocation of β-catenin to the nucleus and its transcriptional activation [117]. The role of ACLY in promoting CRC metastasis was observed in an in vivo model [117]. The ACLY protein can be activated through phosphorylation by different kinases, such as the nucleotide diphosphate kinase, the cAMP-dependent protein kinase and the AKT [118,119,120]. The overexpression of FASN led to increased CRC cell proliferation and metastasis through the AMPK/mTOR pathway. High FASN expression correlated with lymph node metastasis, tumor stage status (TNM) and worse prognosis of CRC patients [121]. SCD could modulate oncogenic pathways, including Akt, AMPK, Wnt and Notch signaling pathways [122,123]. The metabolic axis SCD/ACSLs promotes EMT in CRC cells and is associated with a worse clinical outcome of stage-II CRC patients [110]. Concomitant inhibition of SCD and ACSLs in CRC cells resulted in decreased viability without altering the normal cells [110]. 

**Figure 3:**
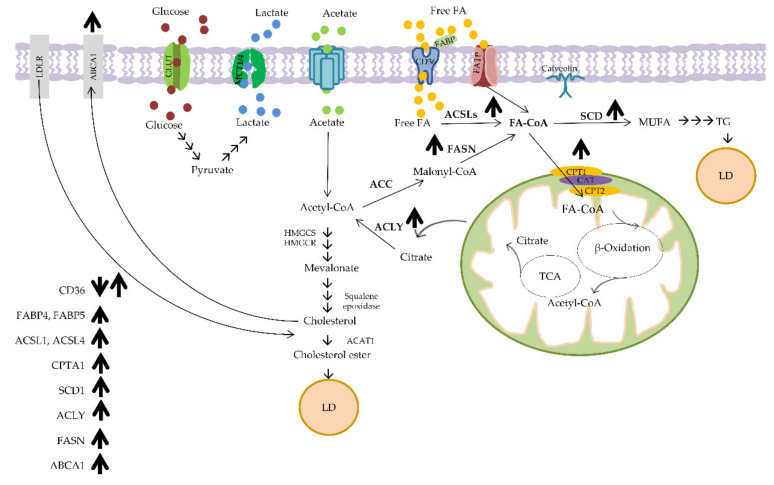
Altered lipid metabolism in CRC. Dysregulated enzymes contributing to abnormal lipogenesis and FAO are clearly represented, with ↑ indicating upregulation, ↓ downregulation and ↓↑ downregulation at an early stage of carcinogenesis and upregulation later in metastatic cancer. TG: triglyceride.

Increased serum cholesterol has been associated with a higher risk of CRC [124,125,126]. Cholesterol is synthesized from acetyl-CoA through a series of reactions mediated by acetyl-CoA acetyltransferase 1 (ACAT1), the 3-hydroxy-3-methylglutaryl-CoA reductase (HMGCR) enzyme, etc. [126]. Stage III–IV CRC patients with a higher expression of HMGCR had a better prognosis. Overexpression of ABCA1, an ATP-binding cassette transporter regulating cellular cholesterol and phospholipid homeostasis, was, however, related to poor CRC prognosis by facilitating tumor growth and caveolin-1-dependent invasiveness [127]. Increased lipid droplet (LD) formation has been found in cancer cells [122]. Downregulation of perilipin 2 (PLIN2), a structure protein involved in the protection of LDs from lipolysis, was found to suppress the proliferation of CRC cells [122]. Alteration in lipid metabolism in CRC is shown in Figure 3.

## 3. Metabolic Pathways Regulating CRC

Colon carcinogenesis is known to be initiated by the accumulation of mutations in Wnt, EGFR, p53 and TGFβ signaling [12]. APC mutation can be detected in the majority of colorectal adenomas (70%). Adenomas develop into carcinomas via the activation of oncogenes (for example, KRAS) and the inactivation of tumor suppressors (such as p53, PTEN and SMAD4) mutations [8,128]. As discussed above, the initiation and development of CRC are accompanied by metabolic alterations. Oncogenic signaling pathways that drive CRC progression regulate metabolic pathways, and the crosstalk between these pathways contributes to the development of CRC [16,129,130,131].

### 3.1. WNT Signaling Influences CRC Metabolism

Abnormal Wnt signaling is recognized as the main trigger and promoter in CRC carcinogenesis [132,133]. Wnt signaling plays important role in intestinal crypt proliferation through setting up the stem cell niche [132]. Abnormal Wnt signaling increases the glucose metabolism via regulating both the pyruvate dehydrogenase kinase 1 (PDK1) and lactate transporter (MCT-1). PDK1 has an inhibitory function on OXPHOs through inhibiting coupling glycolysis to TCA. Wnt/β-catenin induces glutamine uptake and glutaminolysis, which is considered to be managed by c-Myc, one of the downstream target genes of Wnt/β-catenin [134,135]. Wnt/β-catenin also exerts its function on lipogenesis via c-Myc. It induces triglycerides’ phospholipid synthesis, with an increased ratio of unsaturated fatty acids to saturated fatty acids [136]. Two key enzymes involved in lipogenesis, FASN and ACC, could be regulated by Wnt/β-catenin [137]. Furthermore, β-catenin elevates SCD expression indirectly via upregulating the transcription factor SREBP-1 [138].

### 3.2. Oncogenic KRAS Signaling Influences CRC Metabolism

According to a study by Yun et al., mutations in KRAS and BRAF upregulated glucose transporter GLUT-1 and increased glucose uptake in CRC cells, while mitochondrial function and oxidative respiration were not impaired [139]. As suggested by Weinberg et al., oncogenic KRAS requires glutamine-fueled mitochondrial metabolism [140]. When mitochondrial alanine aminotransferase was inhibited, anchorage-independent growth in KRAS-transformed cells was suppressed [140]. During the TCA cycle, there is huge ROS production. Weinberg et al. proposed that oncogenic KRAS increased mitochondrial ROS to control cellular proliferation [140]. It was observed that knockdown of the glutamine transporter in KRAS mutant CRC cells resulted in decreased cell proliferation, migration and invasion, as well as tumor formation and metastasis in vivo [141]. In the study by Toda et al., the effect of oncogenic KRAS on asparagine synthetase (ASNS) was revealed. Mutant KRAS could upregulate ASNS through activating PI3K/AKT/mTOR signaling, thereby promoting CRC cell growth [142].

### 3.3. PI3K/AKT/mTOR Signaling Influences CRC Metabolism

Aberrant PI3K/Akt signaling can be seen in diverse CRC cells. PIK3CA mutations are present in 15% of metastatic CRC cases, and loss of the tumor suppressor PTEN is usually found in 20–40% of CRC patients [143,144,145,146]. Modulation of AKT or AKT downstream effectors such as mTORC1, glycogen synthase kinase 3(GSK3) and the FOXO family of transcription factors regulates metabolism [147]. AKT facilitates glucose uptake through GLUT-1 and GLUT-4 [148]. It was reported that AKT phosphorylates and inactivates the thioredoxin-interacting protein (TXNIP), responsible for endocytosis of the GLUT-1 and GLUT-4 transporters [149]. Furthermore, AKT also increases HK2 activity [149]. PI3K/Akt signaling also affects the metabolism through influencing the downstream transcription factors involved in metabolic control, such as Myc, HIF-1 and SREBP [120,148,150]. PI3K/AKT signaling induces lipogenesis by directly phosphorylating and activating ACLY and increases the level of cytoplasmic acetyl-CoA, a crucial metabolite in carbon and energy metabolism and biosynthetic pathways [119,120,151,152]. In addition, AKT signaling can stimulate lipogenesis through the sterol regulatory element-binding protein (SREBP) family of transcription factors. The downstream target genes of SREBPs are involved in lipid synthesis and NADPH production [153,154,155,156]. AKT signaling has also impact on the other branches of glycolysis other than one-carbon metabolism, glutamine uptake and aspartate synthesis [148]. Akt and downstream effector mTOR1 have been found to promote glucose flux into the oxidative pentose phosphate pathway. mTORC1 also activates this pathway via the activation of SREBP and upregulation of glucose-6-phosphate dehydrogenase, an enzyme that converts glucose-6-phosphate into ribose-5-phosphate on the transcriptional level [157]. Furthermore, mTOR1 activate enzymes important for serine synthesis and one-carbon metabolism, such as MTHFD2 (methylenetetrahydrofolate dehydrogenase) [158]. Glutamine uptake, increased by SLC1A5 transporters, is mediated by Myc, which is stabilized by AKT-mediated inhibition of GSK3-dependent degradation [40,159,160,161]. It is worth mentioning that mTOR1 is controlled through several pathways, including the RAS/MAPK and PI3K/AKT signaling pathways. In line with this, evidence shows that Myc activation is pathway-dependent in cancers; for example, PI3K inhibitors did not change Myc levels in CRC due to the overpowering RAS/MAPK signaling [146,162]. PIK3CA mutations have been found to increase glutamate pyruvate transaminase 2 (GPT2) in colorectal cancer (CRC) cells [163].

### 3.4. P53 Influences CRC Metabolism

TP53, encoding p53 protein, also known as a guardian of genomes, is known for its tumor-suppressive roles [164]. Most cancers, including colorectal cancer, are characterized by mutations in the TP53 gene [165]. Numerous pieces of evidence support the notion that p53 is a major player in controlling the cell cycle, apoptosis and senescence. However, this was challenged by the finding that the knockout of p53-associated genes, such as p21, puma and noxa, showed no increased tumor growth in mice [166,167]. This observation raises the possibility that p53 might exert its tumor-suppressive role through regulating genes involved in metabolism. In agreement with this, various recent findings have shown that p53 functions in metabolic control, contributing to tumor suppression [168,169,170]. Mutant p53 increased glucose uptake by stimulating the translocation of GLUT-1 to the membrane through Rhoa/ROCK signaling [171]. Additionally, p53 induces HK2 enzyme expression, one of the key glycolytic enzymes. In glucose-deprived conditions, mutant p53 negatively regulates AMPK signaling, leading to aerobic glycolysis, lipid synthesis and enhanced cancer cell invasiveness [172]. Eriksson et al. reported that R175H and R273H mutations activated both glycolysis and OXPHOs; on the other hand, R181H, S241F and H179R mutations elevated glycolysis but simultaneously inhibited OXPHOs [173]. Fatty acid synthesis was positively regulated by the mutant p53 through increasing the expression of key genes involved in fatty acid synthesis, such as the FASN gene [174]. Interestingly, the p53 mutant (R273H) has been found to elevate the expression of 17 mevalonate pathway genes involved in cholesterol and isoprenoid synthesis [175,176]. Moreover, p53-deficient CRC cancer cells activated the mevalonate pathway via SREBP2 [177]. The p53-null CRC cell line showed increased glycolysis and lactate compared with the p53 wild type [178]. The role of p53 in the upregulation of crucial players in electron chain transport, such as SCO2 (synthesis of cytochrome c oxidase 2), was reported in CRC cells [179]. In normal conditions, p53 stimulated PDC activity as well as OXPHOs by inhibiting PDK2 is observed in vivo and in vitro, while the mutant p53 upregulated PDK2 in CRC [14,180].

## 4. Tumor Microenvironment (TME)

**Figure 4:**
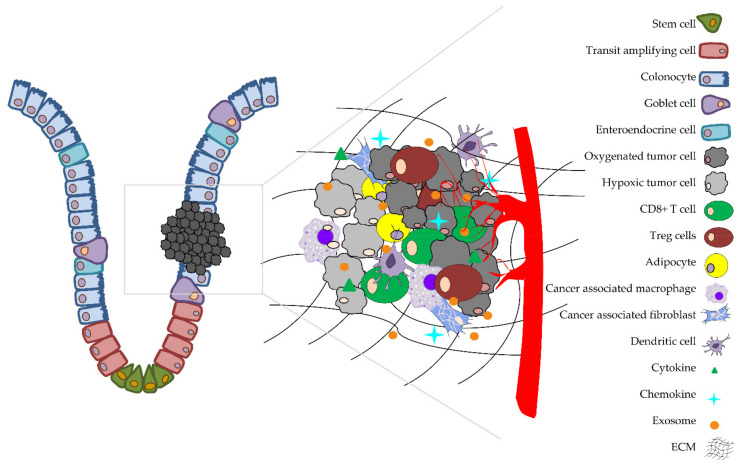
Depiction of tumor mass localized in the colon. Zoomed-in illustration of the tumor microenvironment (TME), including tumor cells, stromal cells and immune cells. Tumor cells with proximity to the blood vessel are marked as oxygenated while distant cells are referred to as hypoxic. The complex networking contains different types of cells, an extracellular matrix (ECM) and molecules such as cytokines, chemokines and microvesicles (such as exosomes).

In recent years, it has become evident that the metabolism of tumor cells is highly affected by the environment in which they reside. This phenomenon shifts our focus from “only tumor cells” to the more complex networking system that includes diverse types of cells and non-cellular components. This conceptual change occurred after metabolic reprogramming in tumor cells and the TME had been recognized. The alteration of the TME is dynamic, aiming to support the high metabolic needs of adjacent tumor cells. Furthermore, metabolic rewiring in the TME contributes to tumor progression and therapy resistance. A group of solid malignant tumors, to which CRC also belongs, contains not only tumor cells but also stromal cells, immune cells and endothelial cells in conjunction with extracellular matrix and signaling molecules, such as chemokines, cytokines and exosomes [181]. ECM is an essential component of the TME. Remodeling of ECM is observed in cancer. It is found that type I collagen, MMP-2 and MMP-9, participating in the degradation and remodeling of the ECM, were upregulated in primary CRC [182]. Tumor cells are bidirectionally connected with both stromal (cancer-associated fibroblasts) and innate (tumor-associated macrophage (TAM)) cells; myeloid-derived suppressor cells (MDSCs); natural killer (NK) cells; dendritic cells; adaptive (immune cells regulatory T (Treg)) cells’ and tumor-fighting effector cells, including cytotoxic CD8 T cells and CD4 T helper cells type 1 (Th1) [181]. The communication between cancer cells and their surrounding microenvironment is shaped to promote cancer cell growth and progression and, by all means, to evade immune surveillance. The most abundant components of the TME are shown in Figure 4.

### 4.1. The Reverse Warburg Effect Reshapes the TME

**Figure 5:**
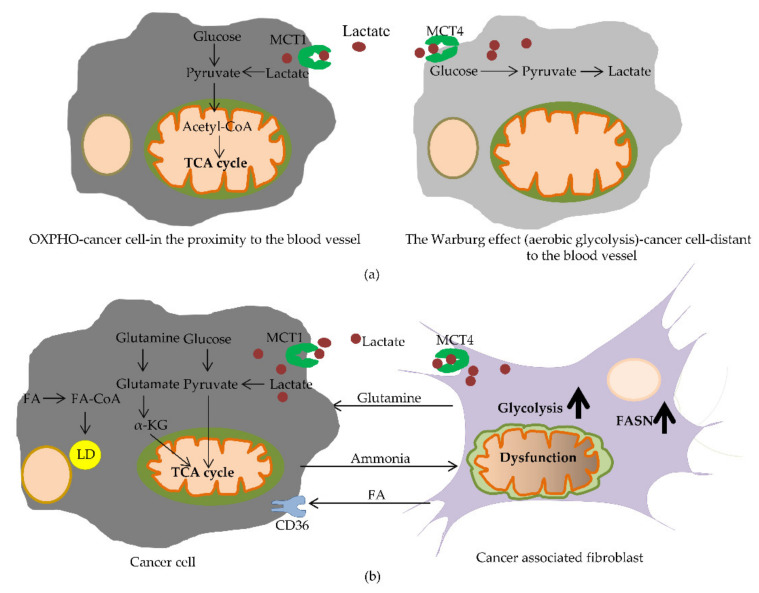
The Warburg effect (aerobic glycolysis) and oxidative phosphorylation (OXPHO) in cancer cells. (**a**) Due to the different localization of cancer cells and consequently different oxygen gradient and supply, glucose can be either converted to lactate or to CO2 through TCA and electron chain transport (TCA cycle, ETC), producing energy in the form of ATP as 2 ATPs and 32–36 ATPs, respectively. (**b**) Metabolic crosstalk between cancer cells and cancer-associated fibroblast (CAFs). Lactate produced in CAFs is transported to the cancer cells via MCT-4 and MCT-1 transporters. In cancer cells, lactate is converted into pyruvate, which is coupled further to the TCA cycle. In addition, increased expression of FASN in CAFs leads to an enhanced level of fatty acids transported into cancer cells through CD36 and used for lipid synthesis, causing increased lipid droplet (LD) formation. Glutamine produced in CAFs affects TCA cycle in cancer cells, while ammonia from cancer cells influences the function of CAFs.

Although the Warburg effect, discussed at the beginning of this review, provided us with new insight into cancer research, this effect was challenged by the finding that OXPHOs were the dominant energy supplier for cervical and breast cancer cells [183,184]. Moreover, this effect did not take into account metabolic communications between cancer and other cells belonging to the tumor microenvironment (TME) [185]. This complexity in the glucose metabolism might explain the reason why glycolysis-targeting therapies are not always effective [186]. Unlike aerobic glycolytic cells, some cancer cells display high rates of OXPHOs. Furthermore, due to the complex tumor heterogeneity, different metabolic phenotypes exist in one tumor mass [187]. Colorectal cancer (CRC) cells were found to rely more on OXPHOs than glycolysis, and they were associated with higher OXPHO rates compared with normal colon cells [188,189]. Numerous studies suggest that the TME plays a significant role in tumorigenesis and therapy resistance [190]. Stromal cells, especially cancer-associated fibroblasts (CAFs), have been found to highly influence the metabolism of cancer, facilitating tumor progression [191,192]. Based on this fact, the new concept “the reverse Warburg effect” was introduced and incorporates the metabolic communication between cancer cells and stromal cells. Simply depicted, secreted reactive oxygen species by cancer cells provoke oxidative stress in the CAFs. As a result, CAFs metabolically shift to aerobic glycolysis, leading to the production of energy-rich metabolites, such as pyruvate, lactate, fatty acids and ketone bodies. This metabolic shift in stromal cells is mediated by the loss of caveolin-1(Cav-1) [193,194,195,196]. The upregulation of mono-carboxylate transporters (MCTs) facilitates the lactate transportation from CAFs to cancer cells [191]. HIF-1α induced in CAFs promotes the synthesis of factors of angiogenesis (VEGF) and aerobic glycolysis by elevating the glycolytic enzymes pyruvate kinase M (PKM1 and PKM2), lactate dehydrogenase A and B and mono-carboxylate transporters [197,198,199]. Nuclear factor κB (NF κB) regulates both the cytokine excretion and metabolism of CAFs [200]. This suggests that the CAF–cancer cell metabolic network may promote tumor growth. The reverse Warburg effect was described in the crosstalk between colon cancer cells and stromal cells in the study of Chekulayev et al. [188]. As previously mentioned, the metabolic program can be strongly affected by intra-tumor heterogeneity [186]. The altered metabolic program further influences the crosstalk between tumor cells and the TME [186]. Changes in the vascular vicinity in the TME during metabolic reshaping lead to altered nutrients and oxygen, with cancer cells closer to the blood vessels showing higher OXPHOs compared with those far away from the blood vessels [201]. The Warburg effect (aerobic glycolysis) and oxidative phosphorylation (OXPHO) in cancer cells are illustrated in Figure 5a. 

### 4.2. Metabolic Changes in CAFs

Fibroblasts are mesenchymal cells that play a role in ECM production during the wound healing process upon injury [202,203,204]. Unlike normal fibroblasts, cancer-associated fibroblasts (CAFs) are closely associated with tumorigenesis [205]. CAFs function in the modulation of ECM and production of pro-tumor signaling molecules, fostering tumor growth, invasion and metastasis [206,207]. In order to adapt to the dynamic TME and availability of nutrients, CAFs undergo metabolic reprogramming [208]. In the study by Zhang et al., the mechanism underlying the metabolic shift in glucose metabolism was suggested. They found that a decrease in the isocitrate dehydrogenase 3 complex α subunit (IDH3α) was related to the metabolic change from oxidative phosphorylation to glycolysis [209]. IDH3α expression was significantly reduced in the CAFs of human CRC compared with normal fibroblasts [209]. Furthermore, CAFs were characterized by an increased level of hexokinase 2 (HK2) and 6-phosphofructokinase-liver type (PFKL), accompanied with increased glucose uptake (GLUT-1) [209]. IDH3α downregulation caused a reduction in α-KG and, consequently, to less conversion into fumarate and succinate and inhibition of prolyl hydroxylase domain protein 2 (PHD2), leading to the stabilization of HIF-1α, which drives glycolysis [209]. As a result of massive glycolysis, increased lactate excretion by the CAFs was detected [209]. Glutamine has been found to display different functions in tumor cells and CAFs. While in cancer cells, glutamine inhibits apoptosis and autophagy accompanied by increased mitochondrial activity, increased autophagy assisted by lower mitochondrial activity has been observed in CAFs [210]. As reported by Gong et al., lipid metabolism reprogramming in CAFs promoted CRC cell migration [208]. It was found that fatty acids, diglycerides (DGs), phosphatidic acid (PA), phosphatidylinositol (PI), lysophosphatidylcholine (LPC) and phosphatidylethanolamines (PE) were significantly upregulated in CAFs compared with normal fibroblasts, accompanied by higher excreted levels of fatty acids and phospholipids [208]. Metabolic reprogramming in CAFs was followed by an increased expression of FASN, and increased fatty acid uptake in CRC cells leads to EMT and metastasis [208]. Metabolic crosstalk between cancer cells and CAFs is summarized in Figure 5b. 

### 4.3. Metabolic Changes in T Cells

T cells play an essential role in the adaptive immune response. Two groups of T cells, namely CD8+ “killer” and CD4+ “helper” T cells, actively participate in immune-mediated cell death [211]. It is known that cancer cells and certain cells of the TME evolve mechanisms to avoid or suppress the immune response through inhibiting the proliferation of helper and killer T cells or by promoting the inflammation-mediated recruitment of immunosuppressive regulatory T cells (Treg) and myeloid-derived suppressor cells (MDSCs) [207]. Recent data have shown that activated T cells participate in glycolysis to meet their needs, unlike naïve T cells that depend on oxidative phosphorylation. Furthermore, activated T cells process glutamine and downregulate FAO [212]. Cancer cells influence these processes through excreting their metabolites into the TME and reshaping the TME by cancer cells, further supporting their rapid growth. The metabolic regulation during T cell activation is mediated by PI3K/Akt/mTOR signaling, resulting in an increased glucose uptake transporter (GLUT-1) [212]. It is found that the 6-phosphofructo-2-kinase/fructose2,6-biphosphatase 3 (PFKFB3) gene is upregulated in immune cells, associated with the increased level of glucose transporter-1 (GLUT-1) [212]. Under hypoxic conditions, T reg cells rely on fatty acid oxidation. During T cell activation, upregulation of SREBP-1 PI3K/Akt signaling results in higher expression of the ACLY and FASN, two key enzymes in FA synthesis. FAO limits the activity of Teff by upregulating PD-1 and carnitine palmitoyl transferase 1A, consequently downregulating INF-γ generation [212].

### 4.4. Tumor-Associated Macrophages (TAMs)

TAMs, an important part of the TME, yield up to 50% of TME components, depending on the type of malignancy [213]. TAMs are able to acquire different phenotypic, metabolic and functional profiles in response to environmental perturbations, ranging from a pro-inflammatory (so-called M1-like) to an anti-inflammatory (so-called M2-like) state [214,215]. As reported by Wei et al., TAMs were found to support CRC growth and metastasis through the induction of EMT via the STAT3/miR-506-3p/FoxQ1 axis, and it was also a potential prognostic marker for CRC patients [216].

### 4.5. Nutrients, Metabolites and Adipocytes Reshape the TME

The TME is reshaped by tumor metabolism. In most tumors, conversion of pyruvate to lactate occurs, which has a toxic effect on cells in higher concentration and acidifies the tumor microenvironment [213]. The more glucose is uptaken, the more lactate is produced, which is then excreted through monocarboxylate transporters (MCT1–4) [214]. High extracellular lactate shows the immunosuppressive function on the cytotoxic T cells by reducing their cytokine production and altering their glycolysis [215]. Lactate suppresses glycolysis in T cells through inhibiting PI3K/AKT/mTOR signaling. Glucose deprivation in the TME can negatively affect the effector functions of T cells and anti-tumor activities of natural killer (NK) cells [216,217,218]. Glycolytic intermediate such as phosphoenolpyruvate maintains Ca^2+^-NFAT signaling and effector functions of T cells [218]. High consumption of glutamine by cancer cells leads to glutamine deprivation in the tumor microenvironment (TME), which impairs the immune function of T cells. A lack of nutrients and constant competition for glucose, glutamine, serine, methionine and tryptophan are related to the immunosuppressive character [122,217]. High HIF-1α expression due to hypoxia affects the anti-tumor function of T cells. Metabolites such as the tryptophan metabolite-kynurenine (produced by tumor cells and TAMs) has been found to suppress CD8 Teff cells via aryl hydrocarbon receptor (AHR) signaling and consequently upregulation of PD-1 [217,219]. In addition, kynurenine mediated AHR signaling in CD4+ T cells promotes their transition into immunosuppressive regulatory T cells [201,217,220]. Adenosine, a breakdown product of adenosine triphosphate (ATP), negatively regulates the cytotoxic functions of T and NK cells while promoting the anti-inflammatory macrophage M2 type [221,222]. As a consequence, the metabolic reprogramming in the TME helps to generate a pro-tumor immune environment and enables tumor cells to evade immune surveillance. 

Deposits of adipose tissue can alter the TME, contributing to CRC cell growth and migration [122,223]. The tumor-promoting effects of adipocytes and fatty acids were reported in CRC; adipocytes increased FAO in CRC cells via upregulating CPT1A, which links the adipocyte-mediated cellular metabolism to the Wnt signaling in CRC cells. CPT1A upregulation induced metabolic changes, promoted β-catenin acetylation and activated the tumor microenvironment rich in adipocytes [224]. Culturing CRC cells with adipocytes increased the lipid content (FA and triacylglycerol) via FABP4, induced epithelial-to-mesenchymal transition (EMT) and activated the AKT pathway in CRC [225]. FABP4, mainly expressed in adipose tissue, has been found to contribute to the metastasis of CRC through the induction of EMT [226].

### 4.6. Microbiota Reshape the Tumor Microenvironment (TME) in CRC

The significance of microbiota and their contributions to the tumorigenesis have been extensively researched recently. Microbiota and their metabolites are closely associated with the function of the intestinal tract, and they can be modified by diet, lifestyle and pathological conditions [227]. Intestinal inflammation and malignancy influence microbiota metabolites and host–microbiota interactions [228,229]. Impairment in microbiota affects the epithelial barrier of the intestine and facilitates the transduction of tumorigenic signaling [230,231]. Intestinal cancer cells together with the components of the TME communicate with microbiota. The emerging role of microbiota in the reshaping of the TME has been reported in several studies [232]. Microbiota can modify the TME through their metabolites, including short-chain fatty acids (SCFAs), lipopolysaccharide and gallic acid; in addition, they also affect tumor immunosuppressive therapy [233,234,235,236]. CRC is characterized by a high presence of Fusobacterium Nucleatum. On the other hand, low levels of some bacteria such as Holdemanella biformis have been detected in CRC [231,237]. These bacteria showed anti-tumor activity through increasing the generation of SCFAs and inhibiting HDAC in adenomas [231,237]. Intestinal flora misbalance is associated with an impaired level of SCFAs and polyamines. SCFAs, including butyrate, acetate and propionate, are known as microbiota metabolites and play a role in maintaining intestinal homeostasis [231,238]. SCFAs, particularly butyrate, enhance the cytotoxic function of CD8 Teff [222,239,240,241]. Additionally, acetate promotes Teff function through epigenetic mechanisms [242]. Butyrate is a very important energy source for colonocytes. It exerts anti-tumorigenic effects via inhibiting histone deacetylase (HDAC) and oncogenic pathways, including Wnt and NfkB signaling [243]. Polyamines could reshape the TME, supporting the immunosuppressive environment via inhibiting production of cytokines such as IFN-Y and TNF, while the opposite effects mediated by polyamines were also observed [244,245]. Spermine contributes to TAM polarization (M2 polarization), while spermidine favors M1 polarization [246]. The diet modulates the efficacy of CRC by reshaping the TME. As reported by Iwamoto et al., the addition of lipids to the TME prevented the tumor inhibitory effect of anti-angiogenic therapy [247,248]. The ketogenic diet, low in carbohydrates but rich in proteins and fat, reduced tumor size in mouse models of colon carcinoma, and recently, it was found that this kind of diet can enhance the anti-cancer therapy efficacy of PI3Kα inhibitors [248,249].

## 5. Targeting Metabolic Crosstalk in CRC

As discussed above, abnormal metabolic pathways, the tumor microenvironment and the intestinal microbiota interact with each other, contributing to CRC progression and metastasis. Thus, they can be considered as potential targets for CRC therapy.

### 5.1. Targeting Metabolites

#### 5.1.1. Glycolysis Inhibitors

Tumor cells take advantage of the “Warburg effect” to adapt to their surrounding microenvironment and achieve a fast fluctuation in energy demand. The inhibition of glycolysis may reduce tumor cell proliferation and metastasis. Xing et al. observed that rapamycin-resistant CRC cells DLD-1 displayed an elevated glycolytic rate with the upregulation of glycolytic enzymes, including hexokinase 2, PKM2 and LDHA, and a combination of the mTOR inhibitor Rapamycin and the glycolysis inhibitor 3,4,5,7-tetrahydroxyflavone resulted in synergistically suppressive effects [250]. In good agreement with this, very recently, a study by Zhai revealed the utility of Oxamate in combination with two other drugs, Metformin and Doxorubicin, in the treatment of CRC. They found that the triple-drug combination triggered autophagy to modulate miR-106a/ULK1 expression [251]. In normal human bronchial epithelial cells (HBEC), Oxamate exhibited much lower toxicity compared to lung cancer cells H1975 with its IC_50_ 96.73 ± 7.60 mmol/L in HBEC and 32.13 ± 2.50 in H1975 [252]. The compound 2-deoxyglucose (2-DG) competitively inhibits the production from glucose to glucose-6-phosphate and is considered a glycolysis inhibitor. It was found that the treatment of 5-fluorouracil-resistant CRC cells with 2-DG downregulated glycolysis-related enzyme expression and reduced the invasive ability of cells, in addition to attenuating EMT-related cytokine secretion and inactivated disintegrin as well as metalloproteinase 10 and metalloproteinase 17 [253]. In several cancer cells, it was observed that 2-DG enhanced radiation- and chemotherapeutic agent-induced toxicity in vitro and in vivo, while protecting normal cells and organs [254]. Several studies revealed the potential of the glycolysis inhibitor (LND) to sensitize tumor cells, including CRC cells, to chemotherapeutic agents. In lung cancer mouse models, LND was found to be able to suppress cancer metastasis to the brain while showing no toxicity, even when mice were administered 50 times the effective cancer inhibitory dose for eight weeks [255]. However, effective activity against CRC refractory to conventional chemotherapy in clinical trials was not observed possibly due to small sample size [256,257,258,259,260].

FOXE1, one of the forkhead box (FOX) transcription factor family members, was found to be a prognostic marker for CRC patients. Knockdown of FOXE1 in CRC cell lines HT29 and SW480 by shRNA remarkably increased cell proliferation, colony formation and promoted glucose consumption, as well as lactate production through the FOXE1/HK2 axis, indicating that targeting FOXE1 could be a promising therapeutic option for CRC [261].

#### 5.1.2. Glutaminolysis Inhibitor

Metabolic rewiring targeting glutaminolysis is considered an important step in providing tumor cells with energy, essential intermediates and redox requirements to facilitate their rapid growth and dissemination. Many studies have demonstrated that glutamine is a major nutrient, redox modulator and essential signal molecule in neoplastic tissues [262,263]. Tumor cells use glutamine to refuel the tricarboxylic acid (TCA) cycle. In this process, glutamine is first converted to glutamate by glutaminases (GLSs), followed by conversion to α-ketoglutarate (α-KG); a TCA cycle intermediate therapeutically targeting glutaminolysis was proven to be an effective strategy to combat cancer [263]. Recently, a study by Schcolnik-Cabera et al. showed that the glutaminolysis inhibitor 6-diazo-5-oxo-l-norleucine (DON) in combination with the glycolysis inhibitor Ionidamine and Orlistat, a drug against obesity, reduced CT26 colon cancer cell viability and cell cycle progression and increased apoptosis, which was associated with decreased oxidative phosphorylation, glycolysis and fuel flexibility [264]. Clinical delivery of DON prodrugs to advanced inoperable solid tumors including CRC in a low daily dosage showed anti-cancer efficacy without obvious toxicity [265]. The newly identified glutaminase (GLSs) inhibitors selenadiazole-derivatives CPD20 and CPD23 exhibited accumulated uptake in tumor cells and improved anti-cancer activity in CRC cells [266]. The combination of the novel GLS inhibitor CB839 with the EGFR monoclonal antibody Cetuximab showed efficacy in Cetuximab-sensitive and -resistant CRC cell models, indicating that patients with refractory metastatic CRC might benefit from this combination therapy targeting both the “fuel” and signaling components required for tumor survival [267]. Additionally, the anti-tumor activity of CB839 in xenograft growth of PIK3CA-mutant CRC cells was enhanced by chemotherapeutic agent 5-fluorouracil without obvious dose-limiting toxicity, indicating the potential of the combination therapy for patients with PIK3CA-mutant colorectal cancer [268]. 

#### 5.1.3. Lipid Metabolism Inhibitors

Abnormal lipid metabolism is recognized as an important metabolic phenotype in CRC cells, involved in a wide range of colorectal carcinogenesis, progressions and metastases [84]. Upregulated lipogenic enzymes are frequently found in patients with aggressive metastatic CRC [269], implying that lipid metabolism-based therapy by targeting these enzymes might be a novel therapeutic option for CRC. 

Fatty acid synthase (FASN) functions as a key element, participating in the de novo biosynthesis of long-chain fatty acids. In patients with CRC, upregulation of FASN is associated with poor clinical outcome [121,270].

Cerulenin, the first identified FASN inhibitor, was originally applied as an antifungal antibiotic agent [271,272]. It was found that Cerulenin suppressed murine CRC cell proliferation, induced apoptosis and reduced liver metastasis of CRC [273]. Additionally, Cerulenin could potentiate Oxaliplatin, a third-generation platinum compound, exhibiting effectiveness in the treatment of CRC. Thus, the combination of Cerulenin and Oxaliplatin with a reduced dose may help to achieve a long-term tolerated chemotherapy for metastatic CRC [274]. Indeed, it was observed that this drug combination enhanced the therapeutic efficacy and reduced neurotoxicity caused by oxaliplatin in CRC cells [275]. 

Luteolin (3,4,5,7-tetrahydroxyflavone), a potent FASN inhibitor found in vegetables, fruits and medicinal herbs, was thought to exert its anti-cancer activity in CRC by the modulation of various tumor signal pathways, including the IGF-1, Keap1-Nrf2-ARE and Wnt-β-catenin pathways [276]. Recently, it was reported that Luteolin was able to inhibit the migration and invasion of CRC in vitro and in vivo by upregulating miR-384 and downregulating PTN expressions [277]. Additionally, Luteolin can be orally or topically administrated without side effects and it may be utilized as an agent that is safer than the conventional anticancer agents [278]. However, an adverse effect of Luteolin on the anticancer ability of Oxaliplatin in the HCT116 CRC cell line was observed [279]. A high dose of Luteolin could affect Oxaliplatin-induced cell cycle arrest and suppress Oxaliplatin-induced p21 expression in HCT116 p53+/+ cells, but not in HCT116 p53−/− cells, suggesting that a high dose of Luteolin may negatively influence Oxaliplatin-based chemotherapy in a p53-dependent manner [279]. 

Evidence supporting the anti-cancer efficiency of FASN inhibitors in CRC is still accumulating. TVB-3664, a novel FASN inhibitor, showed its anti-tumor effect in CRC. Treatment with TVB-3664 reduced tumor growth in CRC cells as well as in a patient-derived xenograft (PDX) model and altered lipid composition of tumors through the regulation of AKT and Erk1/2 oncogenic pathways [280]. Epigallocatechin-3-gallate (EGCG), a bioactive polyphenol found in green tea, could inhibit the activity of FASN [281,282]. A study by Luo demonstrated that EGCG inhibited CRC cell proliferation and migration via downregulation of the transcription factor STAT3 [283]. EGCG was also found to be able to strongly reduce the colorectal liver metastasis in SCID mice [284]. Like other TVB compounds, TVB-3664 is well tolerated in vivo. No drug-related toxicity of TVB-3664 was observed in animal studies [280]. EGCR did not show toxicity in normal embryonic fibroblast cell line (3T3) even at the highest concentration [285] and an in vivo study showed that the oral administration of EGCG to rats for 13 weeks was not toxic at doses up to 500 mg/kg/day [286].

Besides FASN, other enzymes involved in the de novo synthesis of fatty acids also showed tumor-promoting activity in CRC, making them reasonable targets for therapy. For example, ATP citrate lyase (ACLY) facilitated CRC cell metastasis via the stabilization of β-catenin [117], and it could also mediate chemoresistance to SN38 in CRC cells [287]. However, targeting ACLY by using a small-molecule inhibitor GSK165 did not show therapeutic effects, since GSK165 could meanwhile activate the AKT pathway, facilitating tumor cell growth. The inhibition of both ACLY and AKT led to tumor inhibitory effects [287]. The ACC-α inhibitor TOFA triggered CRC cell apoptosis [288]. Besides, TOFA suppresses ovarian cancer cell growth in vitro and in vivo without showing toxicity in multiple mouse organs, including the heart, liver, spleen, lung, kidney and intestinal tissues [289].

Studies demonstrated that not only the enzymes involved in lipid synthesis are targetable, but also the enzymes responsible for lipid transport can be targeted. Overexpression of CD36, an FA transporter (known as fatty acid translocase), led to enhanced proliferation in CRC cells via upregulation of survivin [290]. Additionally, it was observed that the expression of CD36 was enhanced upon inhibition of FASN by TVB-3664 in CRC cells. These results indicate that the suppression of CD36 may improve the effectiveness of FASN-targeted therapy [95]. Cholesterol belongs to the sterol lipid. Targeting cholesterol metabolism by statins, inhibitors of HMG-CoA reductase, showed reduced all-cause mortality and cancer-specific mortality in CRC patients as revealed by a meta-analysis [291].

Selected compounds targeting CRC cell metabolism are summarized in Table 1.

#### 5.1.4. Targeting One-Carbon Metabolism 

One-carbon metabolism is mainly composed of the folate and methionine cycles, generating one-carbon units which are required by tumor cells for nucleotide synthesis, methylation and NADH/NADPH production to maintain their high proliferation [48]. Targeting one-carbon metabolic pathways may inhibit tumor cell growth. In fact, chemotherapeutic agents including methotrexate and 5-FU targeting dihydrofolate reductase (DHFR) and thymidylate synthase (TYMS), two important enzymes involved in one-carbon metabolism, have been clinically applied in treatment of several types of cancer including CRC. However, serious side effects and drug resistance are common problems. Therefore, development of therapeutics targeting other one-carbon pathway enzymes is needed. 

Serine is a main source of one-carbon units. Serine catabolism contains three steps which are mainly catalyzed by SHMT2, MTHFD2 or methylene tetrahydrofolate dehydrogenase 2-like (MTHFD2L) and methylene tetrahydrofolate dehydrogenase 1-like (MTHFD1L) [292]. The mitochondrial isoform of SHMT2 is found to be upregulated in many cancers including CRC, associated with tumor invasiveness and poor prognosis [292]. Recently, a study demonstrated the tumor inhibitory effect of SHIN2, a SHMT inhibitor, in CRC cell line HCT116. It is found that SHIN2 suppressed proliferation of HCT116 in a stereoselective manner, and knockout of SHMT2, but not SHIN1, sensitized HCT116 to SHIN2 treatment [293]. So far, the in vivo anti-tumor effects of SHIN2 in CRC have not yet been well understood. It is observed that SHIN2 synergized methotrexate in a T-cell acute lymphoblastic leukemia mouse model and in a human patient-derived xenograft [293]. 

NADH/NADPH can be produced by one-carbon metabolism, in which MTHFD2 participates. Elevated MTHFD2 expression was found in CRC, related to tumor cell invasiveness [294]. This makes MTHFD2 a potential target for CRC therapy. The first synthetic MTHFD1/2 inhibitor LY345899 has shown its anti-tumor activity in CRC [295]. Treatment of LY345899 led to reduced proliferative rate in primary CRC cells (SW480), its lymph node metastasis (SW620) and a liver metastatic derivative (SW620-LiM2), while no toxicity in the normal colon epithelial cells was observed [296]. In line with this, Ju et al. found that LY345899 inhibited tumor growth and decreased the tumor weight in CRC patient-derived xenograft models without acute or delayed toxicity [297].

Since high serine concentrations were observed in the tumor microenvironment (TME) due to dependency of tumor cells on serine biosynthesis [298], combination of SHMT2 or MTHFD2 inhibitors with other inhibitors of the serine synthesis pathway targeting upstream enzymes such as DHFR, PDGH and NRF2 might lead to more robust anti-cancer activity [292] Additionally, given the fact that serine starvation inhibited tumor growth in mouse models, dietary intervention to restrict serine uptake might be helpful for cancer patients.

Homocysteine, one of the one-carbon metabolism factors, participates in the methionine cycles. Disturbed homocysteine metabolism caused by genetic (mainly mutations and polymorphisms) and epigenetic alterations of the genes (*MTHFR, CBS, MTRR, MTR, MTHFD, BHMT, TYMS* and *TCN 2*), involved in homocysteine metabolism, has been found in many solid tumors including CRC [299]. A dose-response meta-analysis showed that enhanced homocysteine was significantly related to higher risk of digestive tract cancer [300]. However, the precise mechanisms through which the elevated homocysteine contributes to high risk of gastrointestinal cancer have not yet been fully elucidated and the influence of homocysteine on tumor cell proliferation is not yet clear. Plasma homocysteine concentration can be decreased by dietary intervention [301]. For cancer patients, restricted prescription of homocysteine-elevating drugs such as nicotinic acid and fibric acid derivatives together with dietary intervention might be taken into consideration.

### 5.2. Targeting the Tumor Microenvironment (TME)

Tumor cells are strongly affected by their surrounding environment, the tumor microenvironment (TME), which is mainly composed of extracellular matrix, blood vessels, immune cells, fibroblasts and signaling molecules [302]. As previously mentioned, malignant tumors including CRC undergo metabolic rewiring to support their uncontrolled proliferation, metastasis and drug resistance through the activation of TME components and alteration of signal pathways and metabolites [203]. Targeting the dysregulated metabolism in the TME and intervention in their crosstalk may offer therapeutic opportunities in CRC.

#### 5.2.1. Targeting Stroma Components

In the reverse “Warburg effect”, cancer-associated fibroblasts (CAFs), one of the most important stroma components in the TME, undergo aerobic glycolysis and produce and release lactate into the TME to promote tumor oxidative phosphorylation (OXPHO) [303]. This lactate-based tumor feeding by CAFs can be targeted by using a dual MCT-1/MCT-4 inhibitor [304]. Meanwhile, the glutamine-based tumor-feeding by CAFs can also be targeted by the inhibition of glutamine synthetase, a key enzyme for glutamine synthesis. Recently, the inhibition of glycolysis and glutaminolysis has been considered an emerging drug discovery approach to combat cancers, including CRC [305].

#### 5.2.2. Targeting Immune Cells

The function of immune cells in the TME can be largely influenced by metabolic reprogramming during tumor development, leading to impaired anti-tumor immune response [306]. Th immune vulnerability can be targeted. In fact, the immunotherapy has now widely been employed in the field of oncology, which improves the prognosis of cancer [307]. Immune checkpoint proteins PD-1 and CTLA-4 are negative regulators of T cells [308]. The interaction between PD-1 and its ligand PD-L1 affects metabolic pathways, resulting in T-cell exhaustion in tumors [309]. The antitumor immunity enhanced by the CTLA-4 blockade with a monoclonal antibody against CTLA-4 was observed in CRC animal models [310]. The PD-1 blockade by genetic engineering and antibody treatment inhibited not only the CRC cell growth but also their dissemination to the lung [311]. Several monoclonal antibodies targeting PD-1 such as Pembrolizumab (https://www.fda.gov/drugs/drug-approvals-and-databases/fda-approves-pembrolizumab-first-line-treatment-msi-hdmmr-colorectal-cancer, accessed on 9 June 2021) and Nivolumab (https://www.cancer.gov/news-events/cancer-currents-blog/2017/nivolumab-fda-colorectal, accessed on 9 June 2021) were approved for the treatment of metastatic CRC with genetic features such as mismatch repair deficiency or microsatellite instability. 

However, the efficiency of checkpoint immunotherapy can be limited by the dysregulated metabolism in the TME; thus, immunotherapy together with metabolic modulation of the TME may improve the therapeutic effects [312]. Indeed, the combination of the glutaminase 1 (GLS1) inhibitor with PD1 and PD-L1 antibodies enhanced anti-tumor effectiveness by overcoming a metabolic checkpoint blocking T cell activation in CRC [313]. Preliminary analyses demonstrated that the combination of the glutaminase inhibitor CB-839 and the checkpoint inhibitor Nivolumab was well-tolerated [306]. Hence, metabolic inhibition combined with immunotherapy might provide CRC patients with new therapeutic opportunities.

### 5.3. Targeting Microbiota for CRC Prevention and Therapy

As stated above, abnormal microbial metabolites, associated with microbial dysbiosis, affect CRC pathogenesis through various mechanisms, and modulation of microbiota may imply an attractive preventive and therapeutic strategy for CRC. The improvement of dysbiosis through the adjustment of microbiota could be achieved in three ways, namely, dietary intervention, supplementation of probiotics/prebiotics and supplementation of microbial metabolites that are beneficial to health. 

Diet takes part in shaping the microbiome and has a great influence on the risk of CRC. Cumulative evidence has shown that excessive consumption of red meat and processed meat enhances the risk of CRC, while a balanced diet with abundant fiber protected against the initiation of CRC [314]. It has been reported that consumption of emulsifiers could alter the gut microbiota and facilitate malignant intestinal tumor growth [315,316]. On the contrary, several studies suggested the effects of fruits such as apples and berries against CRC [317,318]. These fruits are rich in flavonoids, which reach the colon and interact with the gut microbiota [319,320]. Research data also revealed that fasting inhibited aerobic glycolysis and proliferation in colorectal cancer via suppression of the AKT/mTOR/HIF1α pathway [321].

Probiotics are referred to those living microorganisms that confer health benefits on the host when administered in adequate amounts, while prebiotics are compounds in food that are able to induce the growth and/or activity of beneficial microorganisms, including microbiota [322,323]. Probiotics regulate CRC through various mechanisms, including enhancing the intestinal mucosal barriers, reducing intestinal inflammation, suppressing the activity of pathogenic bacteria and reprogramming the composition of microbiota [324]. Ishikawa et al. observed that Lactobacillus casei administration significantly reduced the atypia of CRC [325]. The treatment of CRC patients undergoing colectomy with probiotics containing Lactobacillus plantarum, Lactobacillus acidophilus and Bifido-bacterium longum improved the integrity of gut mucosal barrier, associated with an enhanced mucosal tight junction protein expression [326]. Probiotic treatment with Bifidobacterium lactis and Lactobacillus acidophilus in CRC patients altered the distinct microbiota signature in tumors [327]. Dietary synbiotics containing prebiotic insulin and probiotics such as L. rhamnosus GG and B. lactis Bb12 reduced cancer risk factors [328]. 

Modulation of microbial metabolites has shown to be effective in CRC prevention and therapy. Recently, butyrate, an SCFA, has gained considerable attention due to its interaction with the gut microbiota in the development of CRC. Butyrate is mostly produced in the colon by intestinal microbiota during fermentation [328]. Fewer butyrate-producing bacteria were detected in the microbiota of CRC patients than in a control group [329]. Numerous studies have demonstrated the anti-cancer activity of butyrate in CRC by influencing various signal pathways, including the NRP-1/VEGF, ERK2/MAPK, Wnt/β-catenin, p53 and epigenetic pathways [330,331,332,333,334]. Additionally, it was found that butyrate exerted its tumor inhibitory effects by upregulating miR-203 and downregulating miR-92a in CRC cells [335,336]. In patient-derived CRC organoid models, Park et al. observed that butyrate enhanced the efficacy of radiotherapy and protected the normal mucosa [337], indicating the potential clinical application of butyrate in combination with radiotherapy in CRC. However, it was found that butyrate fuels the hyperproliferation of MSH2−/− colon epithelial cells and induces colon cancer in APCMin/+MSH2−/− mice [338]. 

Taken together, the modulation of gut microbiota in different ways offers a novel paradigm in CRC prevention and treatment. However, important issues including the safety of probiotics/prebiotics and the paradox of butyrate have not yet been elucidated, posing challenges for the drug development for microbiota-targeted therapies.

### 5.4. Targeting Oncogenic Signaling Pathways

Recent studies demonstrated that certain metabolic enzymes and metabolic signaling pathways can be regulated by oncogenes to provide a survival advantage for tumor cells, enabling them to adapt to nutrient availability in the TME [17,339]. Crosstalk between the oncogenic pathways and metabolic pathways drives CRC progression and development [12]. 

It is recognized that several signal pathways, including Wnt-β-catenin, EGFR/RAS/RAF/MAPK, PI3K, VEGF and p53, are closely associated with the initiation and progression of CRC. A direct link between these pathways and abnormal metabolic events, such as increased glycolysis and glutaminolysis as well as activated phospholipid and FA synthesis, has been established in CRC. Accumulating research data showed that targeting these pathways inhibits CRC cell growth [12,14]. In reality, FDA-approved pharmaceutical drugs targeting EGFR/RAS/RAF/MAPK and VEGF, such as Cetuximab (anti-EGFR monoclonal antibody), Encorafenib (BRAF inhibitor) and Bevacizumab (anti-VEGR monoclonal antibody), have already been successfully applied in the treatment of patients with metastatic CRC.

Clinical trials targeting the Wnt-β-catenin and PI3K/mTOR pathways and oncogenic p53 mutants (https://clinicaltrials.gov/ct2/show/NCT03149679, accessed on 9 June 2021) in CRC are ongoing [340,341]. Oncogenic pathway-based targeted therapy in combination with metabolic-based therapy might provide therapeutic benefits to CRC patients.

## 6. Conclusions

Conceptual progress in the past decade has led to the discovery of two new hallmarks of cancer, namely reprogramming of the energy metabolism and evading the immune destruction of potential generality [19]. Based on these two hallmarks of cancer, immunotherapy with the checkpoint inhibitor has been successfully applied in the treatment of solid tumors, including CRC; on the other hand, many lines of evidence suggest that metabolic pathways, intersecting with oncogenic pathways, the tumor microenvironment and the intestinal microbiota, may be a targetable vulnerability in CRC. Indeed, metabolism-based therapy by using metabolic inhibitors was observed to be effective in the treatment of CRC. However, the development of metabolic therapeutic drugs is still challenging, mainly due to the issues of safety and drug resistance. Combination therapy might reduce side effects of drugs and overcome therapy resistance. 

It is well recognized that obesity and high fat diet increase colorectal cancer risk; thus, targeting lipid metabolism holds more promise in treatment of CRC. Compounds targeting enzymes involved in lipid metabolism merit further investigation. In CRC, upregulation of lipid metabolic enzymes such as FASN and ACC leads to tumor progression and metastasis through activation of oncogenic pathways including Wnt, PI3K/AKT, AMPK/mTOR. Research focusing on the inhibition of de novo lipogenesis and oncogenic pathways will open new avenue for drug development. 

Despite advances in understanding the metabolic network in CRC, many questions about the tumor metabolism and the impact on individual tumor cells have not yet been answered. It is also worthy to note that many research data about tumor metabolic alterations came from cell line models. It is not yet elucidated to which extent tumor cell lines could mimic real tumors, given the complicated tumor microenvironment and the heterogeneity of real tumors. Further translational studies, including those on the application of patient-derived organoid models for metabolic analysis, will broaden our knowledge on the modulation of tumor metabolism, the TME and gut microbiota, which will ultimately lead to the development of more promising strategies for CRC prevention and therapy.

## Figures and Tables

**Table 1:** Compounds targeting glucose, glutamine and lipid metabolism in CRC.

Inhibitor	Main Target Gene/Protein	Mechanism	Toxicity	Reference
Glycolysis inhibitor				
Oxamate	Lactate dehydrogenase (LDHA)	Inhibition of glycolysis; combination of oxamate and	not toxic	[251,252]
		mTOR inhibitor rapamycin showed synergistic effect		
2-Deoxyglucose (2-DG)	Glycolysis-related enzymes	Inhibition of glycolysis and promoting MET to suppress	not toxic	[253,254]
		tumor invasion and metastasis		
Lonidamine (LND)	Mitochondrial pyruvate carrier (MPC)	Sensitizing CRC cells to chemotherapeutic agents	not toxic in mice	[255,256,257,258,259,260]
Glutaminolysis inhibitor				
6-diazo-5-oxo-l-norleucine	Multiple glutamine-utilizing enzymes	Inhibition of glutaminolysis and induction of cellular	not toxic	[264,265]
(DON)		reactive oxygen species		
CB839	glutaminase 1 (GLS1)	Inhibition of glutaminolysis, combination with cetuximab	not toxic	[267,268]
		(anti-EGFR antibody) showed enhanced efficacy		
Lipid metabolism inhibitor				
Cerulenin	Fatty acid synthase (FASN)	Suppression of CRC cell proliferation, induction of	see text	[273,274,275]
		apoptosis and inhibition of metastasis		
3,4,5,7-tetrahydroxyflavone	FASN	Modulation of IGF-1, Keap1-Nrf2-ARE and	not toxic in rats	[276,277,278]
(Luteolin)		Wnt-β-catenin oncogenic pathways		
TVB-3664	FASN and CD36	Inhibition of lipid metabolism and transport;	not toxic	[280]
		regulation of Akt and Erk1/2 oncogenic pathways		
Epigallocatechin-3-gallate	FASN	Inhibited CRC cell proliferation and migration via	not toxic	[281,282,283,286]
(EGCG)		downregulation of the transcription factor STAT3		
GSK165	ATP citrate lyase (ACLY)	Activation of AKT pathway; inhibition of both AKT	N.A.	[287]
		and ACLY resensitized chemotherapy		
TOFA	Acetyl-CoA carboxylase (ACC)	Induction of apoptosis	not toxic in mice	[288,289]

## Data Availability

Not applicable.
